# Prevalence and Genotype Allocation of Pathogenic *Leptospira* Species in Small Mammals from Various Habitat Types in Germany

**DOI:** 10.1371/journal.pntd.0004501

**Published:** 2016-03-25

**Authors:** Anna Obiegala, Dietlinde Woll, Carolin Karnath, Cornelia Silaghi, Susanne Schex, Sandra Eßbauer, Martin Pfeffer

**Affiliations:** 1 Comparative Tropical Medicine and Parasitology, Ludwig-Maximilians-Universität München, Munich, Germany; 2 Institute of Animal Hygiene and Veterinary Public Health, University of Leipzig, Leipzig, Germany; 3 Department of Virology and Rickettsiology, Bundeswehr Institute of Microbiology, Munich, Germany; Institut Pasteur, FRANCE

## Abstract

Small mammals serve as most important reservoirs for *Leptospira* spp., the causative agents of Leptospirosis, which is one of the most neglected and widespread zoonotic diseases worldwide. The knowledge about *Leptospira* spp. occurring in small mammals from Germany is scarce. Thus, this study’s objectives were to investigate the occurrence of *Leptospira* spp. and the inherent sequence types in small mammals from three different study sites: a forest in southern Germany (site B1); a National Park in south-eastern Germany (site B2) and a renaturalised area, in eastern Germany (site S) where small mammals were captured. DNA was extracted from kidneys of small mammals and tested for *Leptospira* spp. by real-time PCR. Positive samples were further analysed by duplex and conventional PCRs. For 14 positive samples, multi locus sequence typing (MLST) was performed. Altogether, 1213 small mammals were captured: 216 at site B1, 456 at site B2 and 541 at site S belonging to following species: *Sorex* (*S*.) *araneus*, *S*. *coronatus*, *Apodemus (A*.*) flavicollis*, *Myodes glareolus*, *Microtus* (*Mi*.) *arvalis*, *Crocidura russula*, *Arvicola terrestris*, *A*. *agrarius*, *Mustela nivalis*, *Talpa europaea*, and *Mi*. *agrestis*. DNA of *Leptospira* spp. was detected in 6% of all small mammals. At site B1, 25 small mammals (11.6%), at site B2, 15 small mammals (3.3%) and at site S, 33 small mammals (6.1%) were positive for *Leptospira* spp. Overall, 54 of the positive samples were further determined as *L*. *kirschneri*, nine as *L*. *interrogans* and four as *L*. *borgpetersenii* while five real-time PCR-positive samples could not be further determined by conventional PCR. MLST results revealed focal occurrence of *L*. *interrogans* and *L*. *kirschneri* sequence type (ST) 117 while *L*. *kirschneri* ST 110 was present in small mammals at all three sites. Further, this study provides evidence for a particular host association of *L*. *borgpetersenii* to mice of the genus *Apodemus*.

## Author Summary

Leptospirosis is one of the most widespread zoonotic diseases and is caused by *Leptospira* spp. Small mammals often serve as maintenance hosts. We evaluated host-pathogen relations for *Leptospira* species and sequence types in different small mammal species captured at three German study sites. *Leptospira* spp. was detected in 5.9% of the captured small mammal species and at all investigated study sites. While a particular host association was observed for *L*. *borgpetersenii* to *Apodemus* spp., a broad host spectrum was detected for *L*. *kirschneri* which was the most common species detected in small mammals from this study. Besides this study is proving first evidence of *L*. *kirschneri* sequence types (ST) 110 and 117 as well as *L*. *interrogans* ST 24 in rodents from Germany. A focal occurrence of *L*. *interrogans* and *L*. *kirschneri* ST 117 was detected. In contrast, *L*. *kirschneri* ST 110 was present in most small mammal species at all three sites.

## Introduction

*Leptospira* spp. are helical-shaped bacteria and form a particular group of causative agents for the zoonotic disease Leptospirosis. *Leptospira* spp. are transmitted through infected urine of small mammals or contaminated water via the direct contact to skin lesions or conjunctivae [1]. Small mammals are described as the most important maintenance reservoirs in nature and thus as an essential vector for several pathogenic *Leptospira* spp. [2, 3, 4, 5]. Leptospirosis is considered the most widespread zoonotic disease worldwide, which is of emerging concern [6]. In the past, Leptospirosis was described to be a disease of occupational risk for harvesters, miners, veterinarians and rodent control workers in Europe [2, 7]. Nowadays, it is increasingly linked to recreational outdoor activities, such as water sports and adventure travels [5, 8]. However, partially due to the broad variety of clinical symptoms, which are nonspecific, the awareness for this disease is not yet present especially in temperate regions [5, 9]. The estimated incidence of clinical cases per year is 0.2 / 100,000 in Germany [10]. Severe cases associated with rats have also been reported [8, 11]. Recently, human cases, which were linked to contaminated water or soil, occurred in Austria [12, 13, 14]. Furthermore leptospirosis outbreaks were reported among triathletes and strawberry harvesters in Germany [15, 16]. The clinical severity of *Leptospira* spp. infection depends on the virulence of the infecting *Leptospira* serovar as well as on the health status of the patient [3]. The taxonomy of *Leptospira* spp. is complex. To date, ten different pathogenic *Leptospira* species with more than 300 serovars, grouped in 20 serogroups are known [17]. The term serogroup is of taxonomic importance and defines groups with antigenetically related serovars. However identical serovars may belong to different *Leptospira* species [2]. Duplex PCR [18] and detailed sequence typing are used for the characterisation of *Leptospira* spp. strains and genotypes while the microscopic agglutination test (MAT) which is important for the categorisation of serovars, is still the gold standard in routine diagnostics [19]. Most commonly, human clinical cases in Europe are caused by *L*. *interrogans* and/or *Leptospira* spp. serovar Grippotyphosa [12, 13, 16, 20]. A recent study from Poland reported also antibody titres in humans against the serovars Australis, Autumnalis, Hebdomadis, Hardjo, Sejroe, Zanoni, Bataviae, Bratislava, Canicola and Grippotyphosa, belonging to 3 species, *L*. *interrogans*, *L*. *borgpetersenii* and *L*. *kirschneri*[21]. Studies from Germany and France reported high prevalences for *Leptospira* spp. in small mammals which are likely responsible for simultaneous human leptospirosis cases [16, 20]. So far there are only a few studies reporting moderate to high prevalences in small mammals, beavers (*Castor fiber*) and wild boars (*Sus scrofa*) from Germany [5, 8, 22]. Little is known about the prevalence and the geographic distribution of pathogenic *Leptospira* spp. in rodent maintenance hosts in Germany. Possible host-pathogen associations were not further determined thus far.

Therefore, this study’s objectives were:

1. Detection of prevalence rates for different pathogenic *Leptospira* spp. in captured small mammals from three selected sites in Germany;
2. Comparison of the detected *Leptospira* spp. and their sequence types (ST) in relation to captured small mammal species and the various study sites.

## Materials and Methods

### Study sites

#### Bavarian Site 1 “Angelberger Forst”, Bavaria (B1)

The site B1, located near Tussenhausen, Bavaria, is a large mixed forest (641 ha). The anthropogenic influence is low thus interaction between wild and domestic animals and humans is limited [23]. Details of this study area have been described before [24, 25].

#### Bavarian Site 2 “Bavarian Forest National Park” (B2)

The second study site B2 was divided in three transects in the “Bavarian Forest National Park” (242,000 ha) from 629 to 1420 m a. s. l. It is also situated in Bavaria and is part of the Bohemian Massif. This National Park and the adjacent Czech Sumava National Park form one of the most homogenous and extensively forested landscapes in Central Europe. The study site is located in a forested montane area and is often frequented by visitors for recreational activities. Further characteristics, GPS coordinates of trapping sites along the transects as well as ecologic properties have been described in detail before [26].

#### Renaturalised area in the city centre of Leipzig, Saxony (S)

The third site (S) is divided in five areas located around the city centre of Leipzig formerly consecutively named from E to I [27]. The study site belongs to a renaturalised location (www.neuseenland.de), which was created out of a former brown coal mining area. Today, the surroundings of this site are the largest recreational area near Leipzig thus many visitors frequent this site. Detailed descriptions about the areas around Leipzig have been published elsewhere [27].

### Sampling of small mammals

Small mammals were trapped with Sherman live animal traps (H. B. Sherman Traps, Inc., Tallahassee, Fla., U.S.A.) in 2012 at site B1, in 2010 at site B2 and from 2010 to 2012 at site S. Traps, baited with apple slices, were placed for at least two consecutive nights per month and site and were checked twice a day. For site B1, 50 traps were set up between July and October in 2012. At site B2 in plot sizes of 18 x 18 m, 16 traps were laid out in a grid. The traps were checked twice on two successive days once a month, from May to October 2010. At site S, small mammals were captured with at least 20 traps per subdivided area per month in August and October in 2010 and from March to June in 2011 (site E, H, I) and further in November 2011 and from March to October in 2012 (site E, F, G, H, I). Collected animals were euthanized in accordance with the German Animal Protection Act and stored at −80°C. Detailed trapping procedures were published elsewhere [26, 27, 28]. Necropsy was carried out with collection of biometric data of all small mammals and kidneys were collected. All small mammals were morphologically identified with taxonomic keys [29]. Further, a conventional PCR targeting the partial mitochondrial *cytochrome b* gene [30] yielding an amplicon of 354 bp was performed with 37 small mammal DNA samples from site B1 (17.1%) and 36 DNA samples from site S (6.7%) including all bycaught small mammal species, which were not rodents, in order to verify morphological identification and to verify successful DNA extraction.

### DNA extraction

Depending on the initial kidney size of the small mammals, kidney samples weighed 0.01–0.05g and were homogenized either by cutting the samples into small pieces each with a sterile scalpel (site B1) or by the use of the Precellys 24 Tissue Homogenizer (Bertin Technologies, Montigny-le-Bretonneux, France) for which 600–800 μl phosphate buffered saline (PBS, pH = 7.2) and 0.6 g of sterile ceramic beads, (PeqLab Biotechnologie GmbH, Erlangen, Germany) sized 1.4 mm, were added to the samples in advance (sites S and B2). DNA was extracted either with the Maxwell 16 LEV Blood DNA Kit (Promega GmbH, Mannheim, Germany) and the corresponding Maxwell 16 System (site B1) or manually with the QIAamp DNA Mini Kit (Qiagen, Hilden, Germany) as recommended by the manufacturers (sites S and B2) after addition of 300 μl lysis buffer and 30 μl proteinase K to each sample and incubation overnight at 56°C in a thermomixer (Eppendorf, Hamburg, Germany). For all samples, quantity and quality of the extracted DNA samples were determined with a spectrophotometer (NanoDrop 2000c respectively NanoDrop ND-1000, Peqlab Biotechnologie GmbH).

### PCR methods

#### Real-Time PCR

For the initial screening of all samples, a real-time PCR targeting a partial sequence (242 bp) of the 32-kDa leptospiral major outer membrane lipoprotein *lipl 32* gene was performed as described [22, 31] by the use of the Mx3000P QPCR System (Agilent Technologies, Santa Clara, C.A., U.S.A.). Samples with a CT-value below 40 were regarded as positive.

#### Duplex PCR

In order to distinguish *L*. *kirschneri* from other pathogenic *Leptospira* spp., samples tested positive by real-time PCR were further determined by a duplex PCR [18]. The duplex PCR was targeting a flagellin-encoding *flaB* gene fragment (563 bp) which exclusively amplifies in *L*. *kirschneri* as well as a preprotein translocase-encoding *secY* gene fragment (285 bp), which also amplifies in several other pathogenic *Leptospira* spp. such as *L*. *interrogans*, *L*. *weilii*, *L*. *noguchii*, *L*. *borgpetersenii*, *L*. *santarosai* and *L*. *meyeri* [5, 18].

#### Conventional PCR

Samples which were positive only for *secY* were further determined by conventional PCR targeting a gene fragment (504 bp) which is encoding for the DNA gyrase subunit B (*gyr B*) [32]. Eight pathogenic *Leptospira* species have been described to be detected by this PCR method: *L*. *interrogans*, *L*. *borgpetersenii*, *L*. *weilii*, *L*. *santarosai*, *L*. *alexanderi*, *L*. genomospecies 1, now known as *L*. *alstonii*, *L*. *noguchii* and *L*. *kirschneri* [32, 33]. PCR products were purified with the NucleoSpin and PCR clean-up kit (MACHEREY-NAGEL, Düren, Germany) according to the manufacturer’s recommendations and subsequently commercially sequenced (Interdisziplinäres Zentrum für Klinische Forschung, Leipzig, Germany) with forward and reverse primers used for PCR amplification. Consensus sequences without primer sequences were analysed and aligned with the MegAlign Pro Software (DNASTAR, Inc., Madison, W.I., U.S.A.), compared to available sequences in the GenBank with BLASTn (National Center for Biotechnology Information, Bethesda MD, USA) and deposited in GenBank under following Acc. No.: KT804429-42.

#### Multi Locus Sequence Typing (MLST)

A set of seven primer pairs was used which amplify different housekeeping gene loci: *glmU*, *pntA*, *sucA*, *fadD*, *tpiA*, *pfkB* and *mreA* [17, 34]. The PCR method was modified for the *fadD* and *glmU* loci as described before [22]. MLST succeeded only for 14 samples tested positive by duplex PCR. Products of all seven primer sets were purified, sequenced and analysed as described above. To receive the particular *Leptospira* sequence type, sequences were trimmed accordingly, and further analysed and compared to sequences at http://leptospira.mlst.net/.

#### Statistical analysis

Confidence intervals (95%CI) for prevalences in small mammals were determined by the Clopper and Pearson method using the Graph Pad Software Prism (Graph Pad Software Inc., San Diego, Ca., USA). Pearson’s chi-squared test was used with a type I error α of 0.05 to test the independence of compared prevalences. Fisher’s exact test was used for small sample sizes tested (n<30). The Bonferroni correction was used to control Pearson’s chi-squared tests computed with multiple values.

#### Ethics statement

The permission of small mammal trapping was granted by the “Landesdirektion Sachsen” at site S (permission number: AZ 36.11–36.45.12/4/12-001) and by the “Regierung von Schwaben” at site B1 (permission number: 55.1-8646-2/30). Permission for small mammal trapping was not needed at site B2 as this study site was investigated for federal concerns by the Bundeswehr. Collected animals were euthanized in accordance with the German Animal Protection Act.

## Results

### Trapping of small mammals

Altogether, 1213 small mammals of eleven different species (737 *M*. *glareolus*, 12 *Microtus agrestis*, 431 either *A*. *flavicollis or A*. *sylvaticus*, one *Sorex coronatus*, seven *Sorex araneus*, four *Crocidura russula*, three *Arvicola terrestris*, two *Talpa europaea*, two *Mustela nivalis*, seven *Apodemus agrarius*, seven *Microtus arvalis*) were captured (Table 1). Altogether, 216 small mammals were captured at site B1 (three species), 456 at site B2 (four species) and 541 at site S (ten species). DNA was extracted from one kidney of all 1213 animals.

**Table 1:** Number of collected small mammal species from each study site with the number of small mammals positive for *Leptospira* spp. as detected by real-time PCR in brackets.

Small mammal family	Small mammal species	Study site			Total
		B1	B2	S	
**Muridae**	***Apodemus flavicollis***	83 (2)	-	164 (13)	**247 (15)**
	*Apodemus* spp.*	-	184 (12)	-	**184 (12)**
	***Apodemus agrarius***	-	-	7 (3)	**7 (3)**
	***Arvicola terrestris***	-	-	3	**3**
**Cricetidae**					
	***Microtus arvalis***	-	-	7 (3)	**7 (3)**
	***Microtus agrestis***	-	11	1	**12**
	***Myodes glareolus***	132 (23)	257 (3)	348 (13)	**737 (39)**
**Soricidae**	***Crocidura russula***	-	-	4	**4**
	***Sorex* spp.**	1	4	3	**8**
**Others**	***Talpa europaea***	-	-	2	**2**
	***Mustela nivalis***	-	-	2 (1)	**2 (1)**
	**Total**	**216 (25)**	**456 (15)**	**541 (33)**	**1213 (73)**

### PCR analysis for *Leptospira* spp.

**Fig 1:**
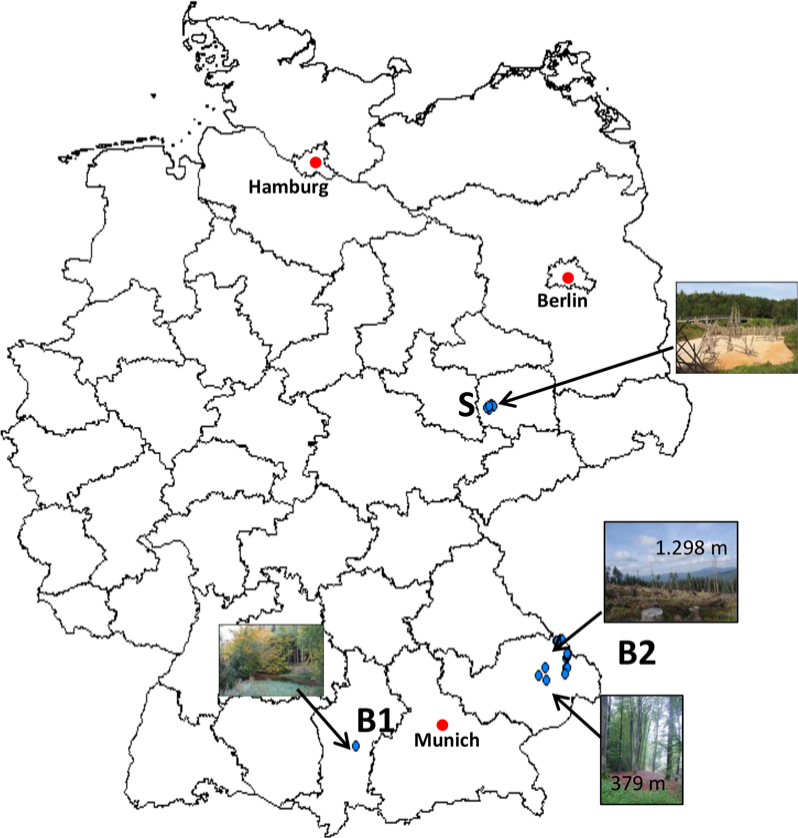
Map of Germany showing the trapping areas (blue dots) of the study sites B1, B2 and S.

From altogether 1213 small mammals, 73 tested positive by real-time PCR (5.9%; 95%CI: 4.7–7.4) (Table 2). Regarding the different sites, 11.6% (95%CI: 8–16.7) of the small mammals from site B1 (n: 25/216), 3.3% (95%CI: 1.9–5.44) from site B2 (n: 15/456) and 6.1% (95%CI: 4.32–8.49) from site S were positive (n: 32/542). Interestingly along the altitude gradient of B2 at two sampling sites at low altitude (both small beech forests at 379 m and 412 m a.s.l., Fig 1) most small mammals of B2 were PCR positive (n: 12). However, *Leptospira* spp. were found up to an altitude of 1.298 m a.s.l. (see Fig 1). From the altogether 73 PCR-positive samples, 67 could be further determined by duplex PCR. The other six samples did not yield any of the two amplicons most likely due to the high CT value achieved by real-time PCR (>39). Fifty-four (80.3%; 95%CI: 69–88.3) of these 67 samples were identified as *L*. *kirschneri*. Four of the other 13 (19.7%; 95%CI: 11.8–31) samples were further determined as *L*. *borgpetersenii*, and 9 as *L*. *interrogans* by conventional PCR of the partial *gyr*B gene and amplicon sequencing.

**Table 2:** Number of the Leptospira species detected in the small mammals per study site*^#^.

Study sites	*Leptospira* species	Small mammal species*^#^						
		*Myodes glareolus*	*Apodemus agrarius*	*Apodemus spp*.	*Apodemus flavicollis*	*Mustela nivalis*	*Microtus arvalis*	Total
**B1**	***Leptospira interrogans***	-	-	-	-	-	-	**-**
	***Leptospira borgpetersenii***	-	-	-	1	-	-	**1**
	***Leptospira kirschneri***	21	-	-	1	-	-	**22**
**B2**	***Leptospira interrogans***	2	-	7	-	-	-	**9**
	***Leptospira borgpetersenii***	-	-	1	-	-	-	**1**
	***Leptospira kirschneri***	1	-	3	-	-	-	**4**
**S**	***Leptospira interrogans***	-	-	-		-	-	**-**
	***Leptospira borgpetersenii***	-	-	-	2	-	-	**2**
	***Leptospira kirschneri***	12	3	-	10	1	2	**28**
**Total**		**36**	**3**	**11**	**14**	**1**	**2**	**67**

*numbers may differ from sum of positive samples detected by real-time PCR as some samples could not be further determined.

^#^from the following small mammal species none was positive for *Leptospira* spp. detected by real-time PCR: *Arvicola terrestris*, *Crocidura russula*, *Talpa europaea*, *Sorex* spp. and *Microtus agrestis*.

*Leptospira borgpetersenii* was exclusively and *L*. *interrogans* was mainly found in *Apodemus* spp. (n: 7/9, 77.8%, 95%CI: 44.3–94.7). Only two *M*. *glareolus* were positive for *L*. *interrogans* (Table 2). *Leptospira kirschneri* was mainly detected in positive small mammals (34 of 36 positive *M*. *glareolus*, 94.4%; 95%CI: 81–99.4; 14 of 25 positive *Apodemus* spp., 56%, 95%CI: 37.0–73.35). *Apodemus agrarius*, *Mustela nivalis* and *Mi*. *arvalis*, though captured in small numbers, showed high prevalences for *L*. *kirschneri* (42.86%; 95%CI: 15.75–75.02; 50%; 95%CI: 9.5–90.5 and 37.5%; 95%CI: 13.5–69.6 respectively (Table 1)). *Leptospira kirschneri* was obtained in four of the five rodent species (*A*. *agrarius*, *Mi*. *arvalis*, *M*. *glareolus*, *A*. *flavicollis*). In contrast, *L*. *interrogans* and *L*. *borgpetersenii* were detected only in *A*. *flavicollis* and *M*. *glareolus*. All other investigated small mammal species were negative.

Whereas most *L*. *kirschneri*-positive rodents were found at site S (n: 27/54, 50.94%; 95%CI: 37.88–63.88), the majority of other pathogenic *Leptospira* spp.-positive samples were found at site B2 (n: 10/13, 76.92%; 95%CI: 49.06–92.5) (χ^2^ = 30.0823; p< 0.00001), at a sampling site 379 m a.s.l.

### Multi Locus Sequence Typing (MLST)

From 67 samples tested positive by duplex PCR, for 14 a complete MLST covering all seven housekeeping genes could be determined for rodents captured at all three study sites (ten *M*. *glareolus*, three *Apodemus* spp. and one *A*. *agrarius*). Altogether sequence types for eleven *L*. *kirschneri* and three *L*. *interrogans* positive samples were detected. All *Leptospira interrogans* positive samples were found to be ST 24 (Table 2). For *L*. *kirschneri* two different sequence types were detected. Four samples were positive for ST 117 and seven for ST 110. While ST 110 could be detected at all three sites, ST 117 was only detected at site S. Here, ST 117 could be detected in *A*. *flavicollis*, *A*. *agrarius* and *M*. *glareolus* whereas ST 110 could only be detected in *M*. *glareolus*. *Leptospira interrogans* ST 24 could be detected in *A*. *flavicollis* and *M*. *glareolus* but exclusively at site B2 (Table 2).

## Discussion

This study focussed on pathogenic *Leptospira* species in small mammals from selected habitats in Germany. Studies on prevalences for *Leptospira* spp. in mammals in Europe are rare and focussed mainly on larger rodent species such as rats (*Rattus norvegicus*) which are considered to be the major source of *Leptospira* infection for humans [34, 35]. High prevalences (20–88%) in *Rattus norvegicus* have been reported from different European countries such as Turkey, France and Denmark [36, 37, 38]. Studies from Germany, Switzerland, the Netherlands, Croatia and Austria showed the occurrence of *Leptospira* spp. in a wide range of different small mammal species including *M*. *glareolus*, *Apodemus* spp., *Mi*. *arvalis*, *Mus musculus*, *Castor fiber* and *Sorex* spp. (2.9–71.4%)[5, 22, 39, 40, 41, 42, 43]. This study’s prevalences show a similar wide range in prevalence regarding the investigated rodent species (5.3–42.9%). The highest prevalence in small mammals was detected at site B1, a forest in southern Bavaria in comparison to the other two study sites. A recent German study showed high prevalences of leptospiral DNA in *Mi*. *arvalis* and *A*. *agrarius* (12–14%) which are supposed to be the most common carrier hosts for *L*. *kirschneri* [5]. In this study the highest prevalence was also found in both of these rodent species for *L*. *kirschneri*. *Leptospira kirschneri* was detected in almost all investigated rodent species (*M*. *glareolus*, *Mi*. *arvalis*, *A*. *flavicollis*, *A*. *agrarius*) with the exception of *Mi*. *agrestis* suggesting that this *Leptospira* species has a broad host range and is well adapted to a number of different small mammal species. Additionally, *L*. *kirschneri* was found in *Mustela nivalis* but not in *Sorex* spp. which therefore may play a subordinate role as maintenance host for *L*. *kirschneri*.

Human leptospirosis case reports caused by *L*. *kirschneri* are scarce. A recent study from Poland, however, reported antibody titres against ten serovars belonging to *L*. *kirschneri*, *L*. *borgpetersenii and L*. *interrogans* in several healthy humans [21]. This argues for asymptomatic infections and a less pathogenic potential than in other pathogenic *Leptospira* species. *Leptospira kirschneri* is known to cause unspecific clinical symptoms in dogs including diarrhoea, lethargy and dehydration [44]. Human cases caused by *L*. *kirschneri* may likewise display such unspecific illness and Leptospirosis may well be overlooked or kept undiagnosed. Nevertheless, this *Leptospira* species should be taken into account as a possible cause of disease in mammals other than dogs and humans.

*Leptospira interrogans* is known to cause severe symptoms such as pneumonia, hepatitis and kidney failure in humans and dogs [45, 46]. The hazardous impact of *L*. *interrogans* to human health was recently described in France in human cases with symptoms such as lumbar myalgia and pneumonia [20]. Moreover unspecific clinical symptoms such as lethargy and fever in humans with previous outdoor activities (e.g. strawberry harvesters, triathletes) were reported in Germany and Austria [12, 13, 15, 16]. High prevalences (33.3–100%) were detected in *Mi*. *arvalis*, *Mus musculus* and *Rattus norvegicus* which occurred sympatrically to human Leptospirosis outbreaks in France and Germany [16, 20]. In former studies *Mi*. *arvalis* was also pointed out to be one of the most important maintenance hosts for *L*. *interrogans* among small mammal species [47, 48]. In the current study, however, this highly pathogenic *Leptospira* species was mostly detected in *Apodemus* spp. and exclusively at site B2, at four locations in a national park, which leads to the assumption that *L*. *interrogans* in contrast to *L*. *kirschneri* (at least of ST 110, see below) rather occurs focally.

*Leptospira borgpetersenii* and in particular serovar Hardjo type Hardjobovis is reported as the most causative leptospiral agent for infertility and abortion in cattle from North America [49]. *Leptospira borgpetersenii* strains were described to be associated with *Mus musculus* [50]. In the present study this *Leptospira* species was detected at all three sites but, exclusively and significantly more often in *Apodemus* spp. than in any other small mammal species which suggests that certain *L*. *borgpetersenii strains* have probably a host preference for the genus *Apodemus* in the investigated habitats.

To the authors’ knowledge, this study is providing first evidence on sequence types of *Leptospira* spp. in rodents from Germany. *Leptospira kirschneri* ST 110 was the most widespread and most common ST in this study. In Germany former studies reported diseases in humans caused by the serogroup Grippotyphosa, declared as “mudfever” and associated with fieldwork activities, such as strawberry harvesting [16]. *Leptospira kirschneri* ST 117 was formerly found in *A*. *agrarius* and *A*. *flavicollis* collected in Croatia [42]. In our study this ST was also found in both of Apodemus species and additionally in *M*. *glareolus*. In Spain *Leptospira interrogans* ST 24was detected in dogs and wild carnivores such as *Vulpes vulpes* showing clinical signs, thus several carnivore species were suggested to be not maintenance but dead end hosts for *Leptospira interrogans* [51]. Further ST 24 was detected in *A*. *flavicollis* from Croatia [42]. In the present study, this sequence type was found in two different rodent species (*M*. *glareolus*, *A*. *flavicollis)*.

It should be taken into account that the comparison of our results between sites and species is limited due to different DNA extraction methods and as the animals examined were not caught in the same years.

In summary, in our study at three sites in Germany pathogenic *Leptospira* spp. were detected in high prevalences in four of five investigated rodent species. Therefore humans could during leisure time activities get into contact with these pathogenic *Leptospira* spp. if respective transmission conditions are optimal. Regarding the *Leptospira* spp. prevalences this study’s results suggest a host preference for *L*. *borgpetersenii* in *Apodemus* spp. Moreover a broad host spectrum was detected for *L*. *kirschneri* which was the most common species detected in this study. Besides this study is proving first evidence of *L*. *kirschneri* ST 110 and 117 as well as *L*. *interrogans* ST 24 in rodents from Germany.

## References

[1] Reis RB, Ribeiro GS, Felzemburgh RD, Santana FS, Mohr S, Melendez AX, et al Impact of environment and social gradient on Leptospira infection in urban slums. PLoS Negl Trop Dis. 2008; 2: e228 10.1371/journal.pntd.0000228 18431445 PMC2292260

[2] Levett PN. Leptospirosis. Clin Microbiol Rev. 2001; 14: 296–326. 11292640 10.1128/CMR.14.2.296-326.2001 PMC88975

[3] Faine S, Adler B, Bolin C, Perolat P, Leptospira and Leptospirosis. 2nd edition Melbourne, Australia, MediSci., 1999; pp. 83–86.

[4] Meerburg BG, Singleton GR, Kijlstra A. Rodent-borne diseases and their risks for public health. Crit Rev Microbiol. 2009; 35: 221–270. 10.1080/10408410902989837 19548807

[5] Mayer-Scholl A, Hammerl JA, Schmidt S, Ulrich RG, Pfeffer M, Woll D, et al Leptospira spp. in Rodents and Shrews in Germany. Int J Environ Res Public Health. 2014; 11: 7562–7574. 10.3390/ijerph110807562 25062275 PMC4143818

[6] Vijayachari P, Sugunan AP, Shriram AN. Leptospirosis: an emerging global public health problem. J Biosci. 2008; 33: 557–569. 19208981 10.1007/s12038-008-0074-z

[7] Bharti AR, Nally JE, Ricaldi JN, Matthias MA, Diaz MM, Lovett MA, Levett PN, et al Leptospirosis: A zoonotic disease of global importance. Lancet Infect Dis. 2003; 3: 757–771. 14652202 10.1016/s1473-3099(03)00830-2

[8] Jansen A, Schöneberg I, Frank C, Alpers K, Schneider T, Stark K. Leptospirosis in Germany 1962–2003. Emerg Infect Dis. 2005; 11: 1048–1054. 16022779 10.3201/eid1107.041172 PMC3371786

[9] Goris MG, Boer KR, Duarte TA, Kliffen SJ, Hartskeerl RA. Human leptospirosis trends, the Netherlands, 1925–2008. Emerg Infect Dis. 2013; 19: 371–378. 10.3201/eid1903.111260 23622144 PMC3647640

[10] Hoffmeister B, Peyerl-Hoffmann G, Pischke S, Zollner-Schwetz I, Krause R, Müller MC, Graf A, Kluge S, Burchard GD, Kern WV, Suttorp N, Cramer JP. Differences in clinical manifestations of imported versus autochthonous leptospirosis in Austria and Germany. Am J Trop Med Hyg. 2010; 83: 326–35. 10.4269/ajtmh.2010.10-0040 20682876 PMC2911179

[11] Roczek A, Forster C, Raschel H, Hörmansdorfer S, Bogner KH, Hafner-Marx A, Lepper H, Dobler G, Büttner M, Sing A. Severe course of rat bite-associated Weil's disease in a patient diagnosed with a new Leptospira-specific real-time quantitative LUX-PCR. J Med Microbiol. 2008; 57:658–63. 10.1099/jmm.0.47677-0 18436602

[12] Windpessl M, Prammer W, Nömeyer R, Dinkhauser P, Wimmer L, Müller P, et al Leptospirosis and renal failure: a case series. Wien klin Wochenschr.2014; 126: 238–242. 10.1007/s00508-014-0501-0 24496714

[13] Radl C, Müller M, Revilla-Fernandez S, Karner-Zuser S, de Martin A, Schauer U, et al Outbreak of leptospirosis among triathlon participants in Langau, Austria, 2010. Wien klin Wochenschr. 2011; 123: 751–755. 10.1007/s00508-011-0100-2 22105111

[14] Hoenigl M, Wallner C, Allerberger F, Schmoll F, Seeber K, Wagner J, Valentin T, Zollner-Schwetz I, Flick H, Krause R. Autochthonous leptospirosis in South-East Austria, 2004–2012. PLoS One. 2014 1 20;9(1): e85974 10.1371/journal.pone.0085974 24465820 PMC3896426

[15] Brockmann S, Piechotowski I, Bock-Hensley O, Winter C, Oehme R, Zimmermann S, et al Outbreak of leptospirosis among triathlon participants in Germany, 2006. BMC Infect Dis. 2010; 10: 91 10.1186/1471-2334-10-91 20380736 PMC2858141

[16] Desai S, van Treeck U, Lierz M, Espelage W, Zota L, Sarbu A, et al Resurgence of field fever in a temperate country: an epidemic of leptospirosis among seasonal strawberry harvesters in Germany in 2007. Clin Infect Dis. 2009; 48: 691–697. 10.1086/597036 19193108

[17] Thaipadungpanit J, Wuthiekanun V, Chierakul W, Smythe LD, Petkanchanapong W, Limpaiboon R, et al A dominant clone of Leptospira interrogans associated with an outbreak of human leptospirosis in Thailand. PLoS Negl Trop Dis. 2007; 1: e56 17989782 10.1371/journal.pntd.0000056 PMC2041815

[18] Gravekamp C, van de Kemp H, Franzen M, Carrington D, Schoone GJ, van Eys GJ, et al Detection of seven species of pathogenic leptospires by PCR using two sets of primers. J Gen Microbiol. 1993; 139: 1691–1700. 8409911 10.1099/00221287-139-8-1691

[19] Bourhy P, Herrmann Storck C, Theodose R, Olive C, Nicolas M, Hochedez P, et al Serovar diversity of pathogenic Leptospira circulating in the French West Indies. PLoS Negl Trop Dis. 2013; 7: e2114 10.1371/journal.pntd.0002114 23516654 PMC3597474

[20] Dupouey J, Faucher B, Edouard S, Richet H, de Broucker C-A, Marié J-L, et al Epidemiological investigation of a human leptospirosis case reported in a suburban area near Marseille. New Microbes New Infect. 2014; 2: 82–83. 10.1002/nmi2.45 25356349 PMC4184663

[21] Wasiński B, Sroka J, Wójcik-Fatla A, Zając V, Cisak E, Knap JP, et al Seroprevalence of leptospirosis in rural populations inhabiting areas exposed and not exposed to floods in eastern Poland. Ann Agric Environ Med. 2012; 19. 22742803

[22] Woll D, Karnath C, Pfeffer M, Allgöwer R. Genetic characterization of Leptospira spp. From beavers found dead in south-west Germany. Vet Microbiol. 2012; 158: 232–234. 10.1016/j.vetmic.2012.02.022 22410308

[23] Forstdirektion Oberbayern-Schwaben: Managementplan zum FFH-Gebiet 7829–301 “Angelberger Forst“, 2004.

[24] Overzier E, Pfister K, Herb I, Mahling M, Böck G Jr, Silaghi C. Detection of tick-borne pathogens in roe deer (Capreolus capreolus), questing ticks (Ixodes ricinus) and ticks infesting roe deer in southern Germany. Ticks Tick Borne Dis. 2013a; 4: 320–328. 10.1016/j.ttbdis.2013.01.004 23571115

[25] Overzier E, Pfister K, Thiel C, Herb I, Mahling M, Silaghi C. Diversity of Babesia and Rickettsia Species in Questing Ixodes ricinus: A Longitudinal Study in Urban, Pasture, and Natural Habitats. Vector Borne Zoonotic Dis. 2013b; 13: 559–564. 23697771 10.1089/vbz.2012.1278 PMC3741418

[26] Thoma BR, Müller J, Bässler C, Georgi E, Osterberg A, Schex S, et al Identification of factors influencing the Puumala virus seroprevalence within its reservoir in a montane forest environment. Viruses. 2014; 6: 3944–3967. 10.3390/v6103944 25341661 PMC4213572

[27] Silaghi C, Hamel D, Thiel C, Pfister K, Pfeffer M. Spotted fever group rickettsiae in ticks, Germany. Emerg Infect Dis. 2011; 17: 890–892. 10.3201/eid1705.101445 21529404 PMC3321775

[28] Obiegala A, Pfeffer M, Pfister K, Tiedemann T, Thiel C, Balling A, et al Candidatus Neoehrlichia mikurensis and Anaplasma phagocytophilum: prevalences and investigations on a new transmission path in small mammals and ixodid ticks. Parasit Vectors. 2014; 7: 563 10.1186/s13071-014-0563-x 25465390 PMC4264555

[29] Stresemann E., 1989, Exkursionsfauna von Deutschland, Wirbeltiere, Volume 3 Heidelberg: Spektrum Akademischer Verlag, Gustav Fischer.

[30] Parson W, Pegoraro K, Niederstätter H, Föger M, Steinlechner M. Species identification by means of the cytochrome b gene. Int J Legal Med. 2000; 114: 23–28. 11197623 10.1007/s004140000134

[31] Stoddard RA, Gee JE, Wilkins PP, McCaustland K, Hoffmaster AR. Detection of pathogenic Leptospira spp. through TaqMan polymerase chain reaction targeting the LipL32 gene. Diagn Microbiol Infect Dis. 2009; 64:247–255. 10.1016/j.diagmicrobio.2009.03.014 19395218

[32] Slack AT, Symonds ML, Dohnt MF, Smythe LD. Identification of pathogenic Leptospira. species by conventional or real-time PCR and sequencing of the DNA gyrase subunit B encoding gene. BMC Microbiol. 2006; 6: 95 17067399 10.1186/1471-2180-6-95 PMC1630700

[33] Smythe L, Adler B, Hartskeerl RA, Galloway RL, Turenne CY, Levett PN, International Committee on Systematics of Prokaryotes Subcommittee on the Taxonomy of Leptospiraceae. Classification of Leptospira genomospecies 1, 3, 4 and 5 as Leptospira alstonii sp. nov., Leptospira vanthielii sp. nov., Leptospira terpstrae sp. nov. and Leptospira yanagawae sp. nov., respectively. Int J Syst Evol Microbiol. 2013; 63: 1859–62. 22984140 10.1099/ijs.0.047324-0

[34] Maiden MC, Bygraves JA, Feil E, Morelli G, Russell JE, Urwin R, et al Multilocus sequence typing: a portable approach to the identification of clones within populations of pathogenic microorganisms. Proc Natl Acad Sci USA. 1998; 95: 3140–5. 9501229 10.1073/pnas.95.6.3140 PMC19708

[35] Tucunduva de Faria M, Athanazio DA, Gonçalves Ramos EA, Silva EF, Reis MG, Ko AI. Morphological alterations in the kidney of rats with natural and experimental Leptospira infection. J Comp Pathol. 2007; 137: 231–8. 17996544 10.1016/j.jcpa.2007.08.001

[36] Sunbul M, Esen S, Leblebicioglu H, Hokelek M, Pekbay A, Eroglu C. Rattus norvegicus acting as reservoir of leptospira interrogans in the Middle Black Sea region of Turkey, as evidenced by PCR and presence of serum antibodies to Leptospira strain. Scand J Infect Dis. 2001; 33: 896–8. 11868761 10.1080/00365540110076796

[37] Aviat F, Blanchard B, Michel V, Blanchet B, Branger C, Hars J, et al Leptospira exposure in the human environment in France: a survey in feral rodents and in fresh water. Comp Immunol Microbiol Infect Dis. 2009; 32: 463–476. 10.1016/j.cimid.2008.05.004 18639932

[38] Krøjgaard LH, Villumsen S, Markussen MDK, Jensen JS, Leirs H, Heiberg A-C. High prevalence of Leptospira spp. in sewer rats (Rattus norvegicus). Epidemiol Infect. 2009; 137: 1586–1592. 10.1017/S0950268809002647 19393116

[39] Kocianova E, Kozuch O, Bakoss P, Rehacek J, Kovacova E. The prevalence of small terrestrial mammals infected with tick-borne encephalitis virus and leptospirae in the foothills of the southern Bavarian forest. Germany Appl Parasitol. 1993; 34: 283–290. 8298661

[40] Adler H, Vonstein S, Deplazes P, Stieger C, Frei R. Prevalence of Leptospira spp. in various species of small mammals caught in an inner-city area in Switzerland. Epidemiol Infect. 2002; 128: 107–109. 11895085 10.1017/s0950268801006380 PMC2869789

[41] Hartskeerl PA, Terpstra WJ. Leptospirosis in wild animals. Vet Q. 1996; 18: 149–150. 8933702

[42] Turk N, Milas Z, Margaletic J, Staresina V, Slavica A, Riquelme-Sertour N, et al Molecular characterization of Leptospira spp. strains isolated from small rodents in Croatia. Epidemiol Infect. 2003; 130: 159–166. 12613757 10.1017/s0950268802008026 PMC2869950

[43] Schmidt S, Essbauer SS, Mayer-Scholl A, Poppert S, Schmidt-Chanasit J, Klempa B, et al Multiple infections of rodents with zoonotic pathogens in Austria. Vector Borne Zoonotic Dis. 2014; 14: 467–475. 10.1089/vbz.2013.1504 24915446 PMC4098071

[44] Greenlee JJ, Bolin CA, Alt DP, Cheville NF, Andreasen CB. Clinical and pathologic comparison of acute leptospirosis in dogs caused by two strains of Leptospira kirschneri serovar grippotyphosa. Am J Vet Res. 2004; 65: 1100–1107. 15334844 10.2460/ajvr.2004.65.1100

[45] Schulze MH, Raschel H, Langen HJ, Stich A, Tappe D. Severe Leptospira interrogans serovar Icterohaemorrhagiae infection with hepato‐renal‐pulmonary involvement treated with corticosteroids. Clin Case Rep. 2014; 2: 191–196. 10.1002/ccr3.91 25614810 PMC4302624

[46] Geisen V, Stengel C, Brem S, Müller W, Greene C, Hartmann K. Canine leptospirosis infections–clinical signs and outcome with different suspected Leptospira serogroups (42 cases). J Small Anim Pract. 2007; 48: 324–328. 17490440 10.1111/j.1748-5827.2007.00324.x

[47] Kuiken T, van Dijk JE, Terpstra WJ, Bokhout BA. The role of the common vole (Microtus arvalis) in the epidemiology of bovine infection with Leptospira interrogans serovar hardjo. Vet Microbiol. 1991; 28: 353–361. 1949549 10.1016/0378-1135(91)90070-v

[48] Treml F, Pejcoch M, Holesovska Z. Small mammals—natural reservoirs of pathogenic leptospiroses. Vet Med Czech. 2002; 47: 309–311.

[49] Grooms DL. Reproductive losses caused by bovine viral diarrhea virus and leptospirosis. Theriogenology. 2006; 66: 624–628. 16716386 10.1016/j.theriogenology.2006.04.016

[50] Perez J, Brescia F, Becam J, Mauron C, Goarant C. Rodent abundance dynamics and leptospirosis carriage in an area of hyper-endemicity in New Caledonia. PLoS Negl Trop Dis. 2011; 5: e1361 10.1371/journal.pntd.0001361 22039557 PMC3201910

[51] Millán J, Candela MG, López-Bao JV, Pereira M, Jiménez MÁ, León-Vizcaíno L. Leptospirosis in wild and domestic carnivores in natural areas in Andalusia, Spain. Vector Borne Zoonotic Dis. 2009; 9: 549–554. 10.1089/vbz.2008.0081 18973450

